# Fast intelligent cell phenotyping for high-throughput optofluidic time-stretch microscopy based on the XGBoost algorithm

**DOI:** 10.1117/1.JBO.25.6.066001

**Published:** 2020-06-03

**Authors:** Wanyue Zhao, Yingxue Guo, Sigang Yang, Minghua Chen, Hongwei Chen

**Affiliations:** Tsinghua University, Beijing National Research Center for Information Science and Technology, Department of Electronic Engineering, Beijing, China

**Keywords:** time-stretch microscopy, imaging cytometry, automatic cell detection, machine learning

## Abstract

**Significance:** The use of optofluidic time-stretch flow cytometry enables extreme-throughput cell imaging but suffers from the difficulties of capturing and processing a large amount of data. As significant amounts of continuous image data are generated, the images require identification with high speed.

**Aim:** We present an intelligent cell phenotyping framework for high-throughput optofluidic time-stretch microscopy based on the XGBoost algorithm, which is able to classify obtained cell images rapidly and accurately. The applied image recognition consists of density-based spatial clustering of applications with noise outlier detection, histograms of oriented gradients combining gray histogram fused feature, and XGBoost classification.

**Approach:** We tested the ability of this framework against other previously proposed or commonly used algorithms to phenotype two groups of cell images. We quantified their performances with measures of classification ability and computational complexity based on AUC and test runtime. The tested cell image datasets were acquired from high-throughput imaging of over 20,000 drug-treated and untreated cells with an optofluidic time-stretch microscope.

**Results:** The framework we built beats other methods with an accuracy of over 97% and a classification frequency of 3000  cells/s. In addition, we determined the optimal structure of training sets according to model performances under different training set components.

**Conclusions:** The proposed XGBoost-based framework acts as a promising solution to processing large flow image data. This work provides a foundation for future cell sorting and clinical practice of high-throughput imaging cytometers.

## 1 Introduction

Nowadays, imaging cytometry is increasingly considered a solution to the detection of cells or particles without demanding a biomarker. Continuous ultrafast imaging enabled by optical time-stretch technology achieves unprecedented imaging speed of millions of frames per second.^1^ The high-throughput label-free imaging cytometer based on optofluidic time-stretch technology combines time-wavelength-space mapping using spatial and temporal dispersion with high-speed single-pixel detection.^2^ It has facilitated circulating tumor cells detection at single-cell sensitivity from abundant cells by capturing cell images rapidly, which is a proper solution for the highly sensitive detection of rare cells.^3^ Researchers have explored extensively to further improve the performance of optofluidic time-stretch microscopy, such as having a higher resolution,^4^ a lower system cost,^5^^,^^6^ and an application to broader scenarios.^7^^–^^9^ However, high-throughput time-stretch imaging cytometry still suffers from the analysis of mass amounts of cell images. A high processing cost would prevent further developments and clinical applications of time-stretch flow cytometry, such as cell sorting.

Machine learning is a powerful tool for finding patterns and identifying different cell types from large-scale data, providing a nonmanual method to process biomedical information.^10^^–^^12^ Many different machine learning approaches to phenotype cell images obtained by optofluidic time-stretch microscopy have been developed. Nitta et al.^13^ proposed a method of cellular deep neural network that classifies cells accurately to sort cells on-chip according to their images. Kobayashi et al.^7^ applied the support vector machines (SVM) classification algorithm to distinct drug-treated and untreated cells properly. Jiang et al.^8^ chose logistics regression (LR) to identify aggregated platelets in blood. Meanwhile, most of these previous studies have overlooked the processing speed of the algorithms while focusing on classification accuracy. As large amounts of cells are continuously imaged by the cytometer, a cell classification algorithm with accuracy and celerity is highly demanded.

However, the LR, SVM, and deep neural network are all missing the standard. LR underfits complicated models due to its linearity; the complexity of SVM models explodes with larger sample sets; and deep neural network with multilayer convolution operation results in high computational complexity. A classification algorithm with low computation cost and sufficient fitting capability is required. Boosting is a tool of massively parallel simple weak classifiers that operates fast and from a complicated model. It appreciates plain features. A mutual characteristic of the images of flowing cells is their regularity containing predictable contents and little impurities or noise, which implies extractable and explicable features.^14^ Therefore, boosting may be the solution to the problem. Here, we introduce a recent boosting algorithm for big data processing called XGBoost.^15^ It is currently one of the best open-source boosted tree toolkits and has shown outstanding performance in many standard classification tasks. Soon after XGBoost was raised, 17 of the 29 champions of the 2015 Kaggle data challenges used the XGBoost method, which beat neural networks with 11 champions.^16^ Moreover, as cell libraries are constructed automatically, the noise in images among them affect learning-based classification models severely. To enhance the trained model’s robustness, we adopt density-based clustering algorithms to detect and remove the noise samples in advance.

In this paper, we implement a framework based on XGBoost for the problem of fast phenotyping of cells in high-throughput optofluidic time-stretch microscopy. The phenotyping consists of detection of outlier samples, extraction of fused features, and XGBoost classification. It is tested on a collection of over 20,000 flow cell images obtained by an optofluidic time-stretch microscope.

## 2 System Overview

### 2.1 Imaging System Setup

**Fig. 1:**
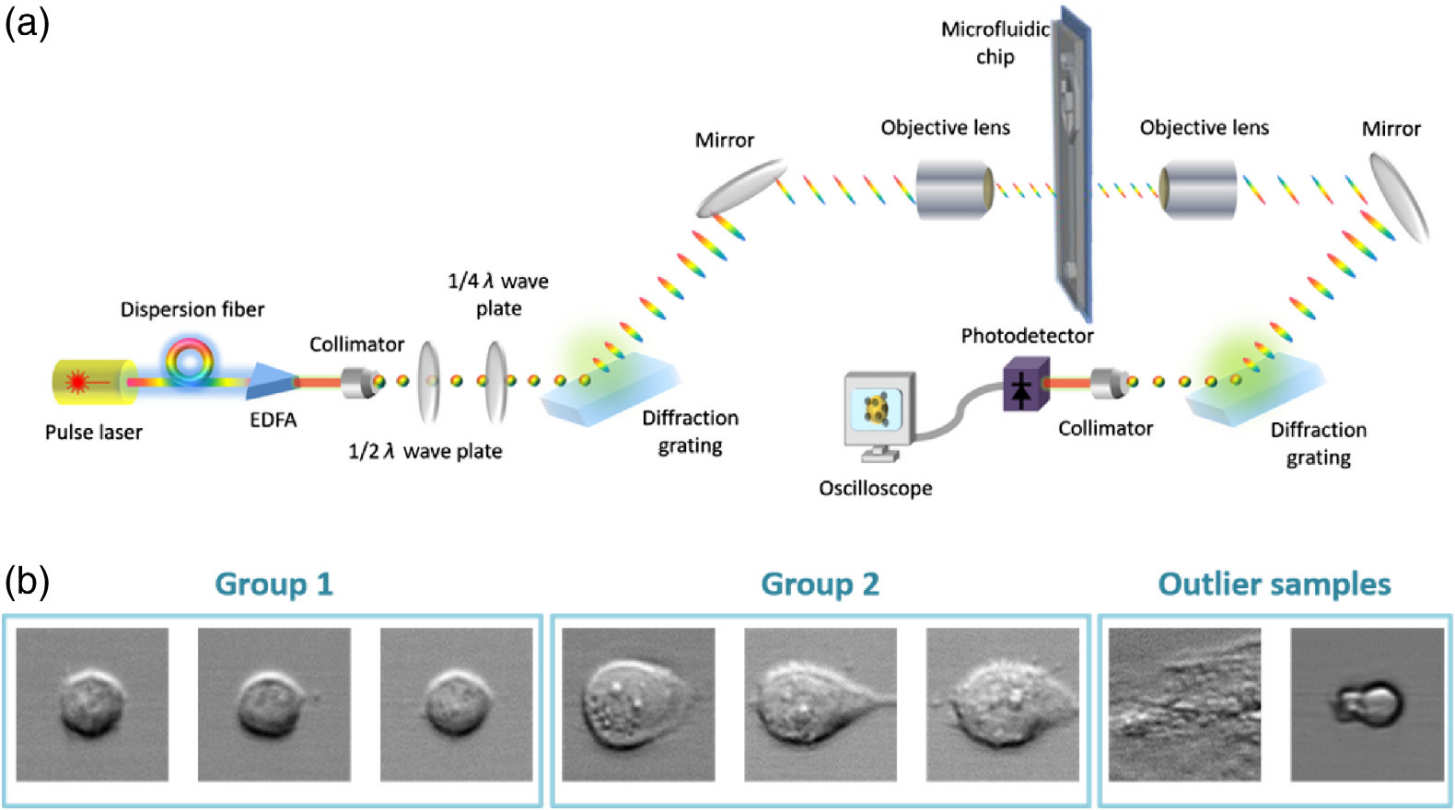
(a) Optofluidic time-stretch imaging cytometer system setup. EDFA, erbium-doped fiber amplifier. (b) Images of drug-treated (group 2) and untreated (group 1) MCF-7 cells under our optofluidic time-stretch microscope (flowing at a speed of 10  m/s). Outlier samples are the images of noise samples such as bubbles and broken cells.

The proposed optofluidic imaging system utilizes a time-stretch imaging structure to break the frame rate limitation of complementary metal-oxide-semiconductor or charge-coupled device, which are commonly used in imaging flow cytometers. In the optofluidic time-stretch imaging cytometer [Fig. 1(a)], a femtosecond pulse laser having a 780-nm center wavelength, 40-nm bandwidth, and 75-MHz pulse repetition rate is used as a light source. The laser pulses emitted from the laser are first dispersed in the time domain by a dispersion fiber (−240  ps/nm dispersion) and then dispersed in the space domain by a diffraction grating with a grating constant of 1200  lines/mm such as rainbow flashes. Then, the dispersed laser pulses are focused by an objective lens (NA=0.6) to illuminate the target cells flowing in the microfluidic chip, and the spatial information of the cells are focused onto the pulses. We employ hydrodynamic focusing in the microfluidic chip to sequence and focus cells during imaging. The cross-section size of the main microchannel is 100-μm wide and 44-μm high. The total flow rate including the sheath flow and the sample flow is 2.75  ml/min, resulting in a flow rate of about 10  m/s. The laser pulses carrying the cellular image information are transmitted through another objective lens, then spatially recombined by another diffraction grating to a single pulse laser beam, and detected by a high-speed photodetector with a bandwidth of 12 GHz. A high-speed oscilloscope with a bandwidth of 16 GHz and a sampling rate of 50  G points/s collects the signal from the photodetector to digitize the cell image information contained in the pulses. Finally, the two-dimensional (2-D) images of the flowing cells [Fig. 1(b)] are obtained by digitally stacking each pulse with cell image information.

### 2.2 Cell Sample Treatment

Multivariate single-cell imaging is effective for evaluating drug-induced phenotypic variations in gene expression, protein localization, and cytoskeletal structure.^17^ Cell responses to drugs for unknown compounds can be correctly predicted accordingly. The optofluidic time-stretch imaging cytometer is capable of acquiring bright-field images of numerous drug-treated and untreated cells by time-stretch microscopy with a high throughput. And the acquired label-free cell images are identified by machine learning through their morphological differences, which are too subtle to detect directly.

Here, we use drug-treated and untreated human breast cancer adenocarcinoma cell line MCF-7 (DS Pharma Biomedical) for cellular drug response detection as sample cells [Fig. 1(b)].^7^ The cells were maintained in Dulbecco’s modified Eagle medium supplemented with 10% fetal calf serum and 1% penicillin–streptomycin at 37°C and 5% ${\mathrm{CO}}_{2}$. Paclitaxel is an food and drug administration approved anticancer drug that is dissolved in dimethyl sulfoxide in powder form at a stock concentration of 1 mM. The cells were incubated with paclitaxel one day after the inoculation, harvested at two intervals (12 and 24 h), suspended in culture medium by trypsinization, and imaged with our time-stretching optofluidic microscope. To ensure reliable single-cell image acquisition in each image, a low cell concentration suspension of about 100  cells/ml was used for the samples. As both the drug-treated and untreated cell suspension are imaged [Fig. 1(b)], it is essential to identify each drug-treated and untreated cell image from the large dataset for further drug-response study.

### 2.3 Structure of Proposed Algorithm

**Fig. 2:**
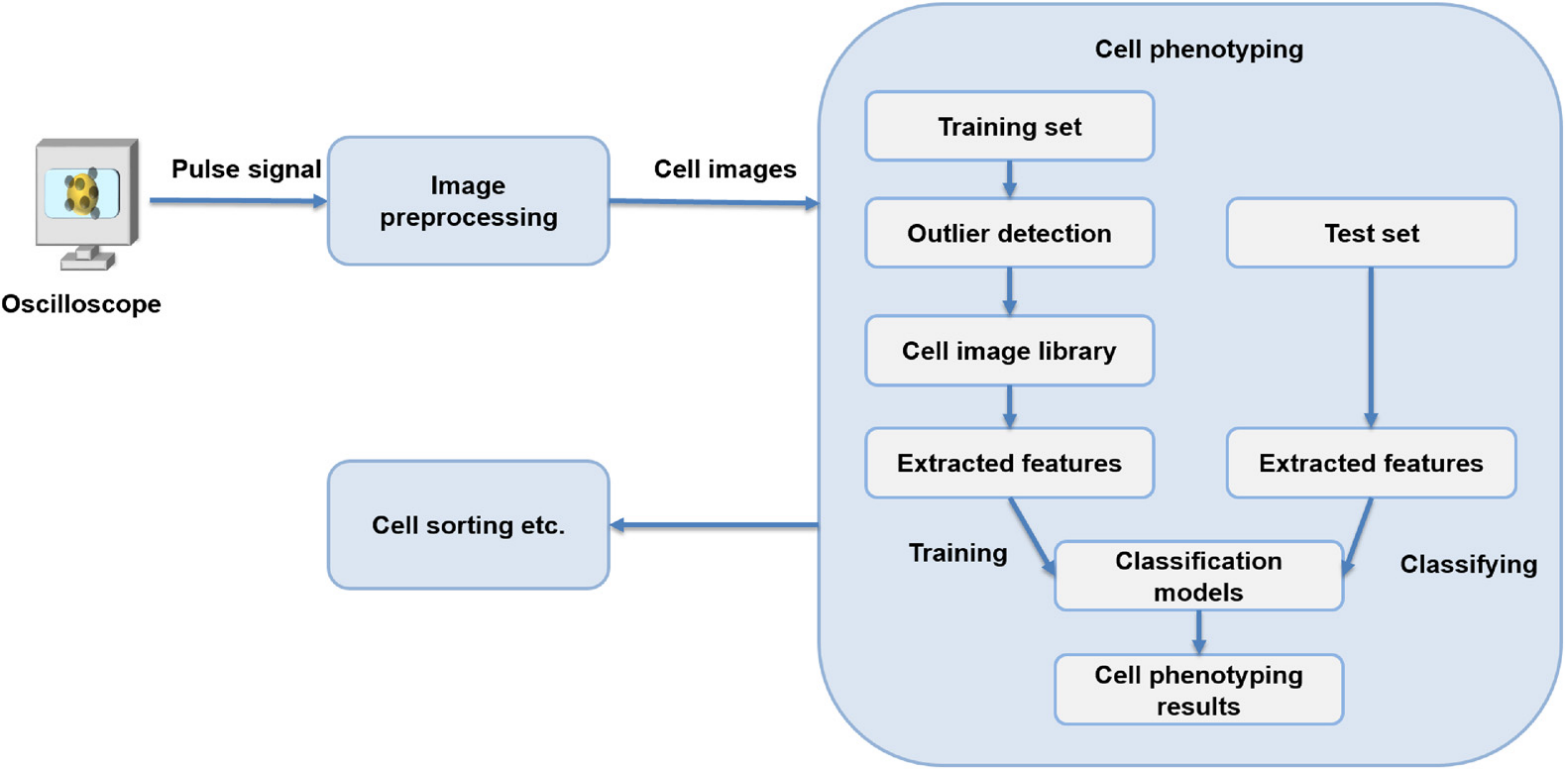
Flowchart of cell phenotyping steps.

As the imaging frame rate of the flow cytometer imaging system reaches the laser repetition rate, which is 75 MHz, 12 GB of cell images are produced by an oscilloscope (Agilent DSO91204A Infiniium) in txt format under 8-bit quantization. Highly accurate image classification can help set similar cells apart, but equally important is the speed of image recognition to enable continuous operation of the phenotyping system with such a mass production of images. Here, we construct a recognition method mainly consisting of three steps: outlier detection, feature extraction method, and classification algorithm. A total of 21,237 cell images (9267 drug-treated MCF-7 cell images and 11970 untreated MCF-7 cell images) are obtained by time-stretch microscopy with a high throughput (up to 10,000  cells/s). The raw data are reconstructed and processed by Python running on a MacBook Air with a CPU frequency of 1.80 GHz and 8G memory. The flowchart of our phenotyping steps is shown in Fig. 2.

The pulses containing cell images collected by the oscilloscope are stacked into 2-D images by an image preprocessing module. In the cell phenotyping module, experiments are designed to construct our framework. Part of the sample images constitutes a training set. The first part of our experiment shows how to build adequate cell image libraries by adjusting the composition of training sets and removing outlier samples with clustering algorithms. The second part selects the best-fit features to extract from both high- and low-dimensional features. The third part proves the efficiency of the XGBoost classification model by evaluating its performance against three other models. Furthermore, the generalization ability of the constructed XGBoost phenotyping framework is tested on another cell image database. The final output of the classification results can be used for subsequent cell sorting.

## 3 Outlier Detection

As can be seen from Fig. 1(b), a classification model should be trained to distinguish cell sample group 1 from group 2. As cells are imaged at a high throughput, cell libraries are constructed automatically by trigger or segmentation algorithms from large raw data. Since it is impossible to distinguish noise images (bubbles, broken cells, etc.) manually in large image libraries, these noise samples would affect learning-based classification models severely if the models are fit according to obtained cell image libraries directly. Therefore, the noise samples, also called outlier samples, ought to be removed from the training set in advance to prevent the negative impact on model training. Clustering methods deal with separating samples into different clusters based on their similarity or density without prior knowledge or training. This section provides a comparison of three density-based clustering algorithms by running a standalone application on each of the algorithms: density-based spatial clustering of applications with noise (DBSCAN), density-based clustering (DENCLUE), and local outlier factor (LOF).

**Fig. 3:**
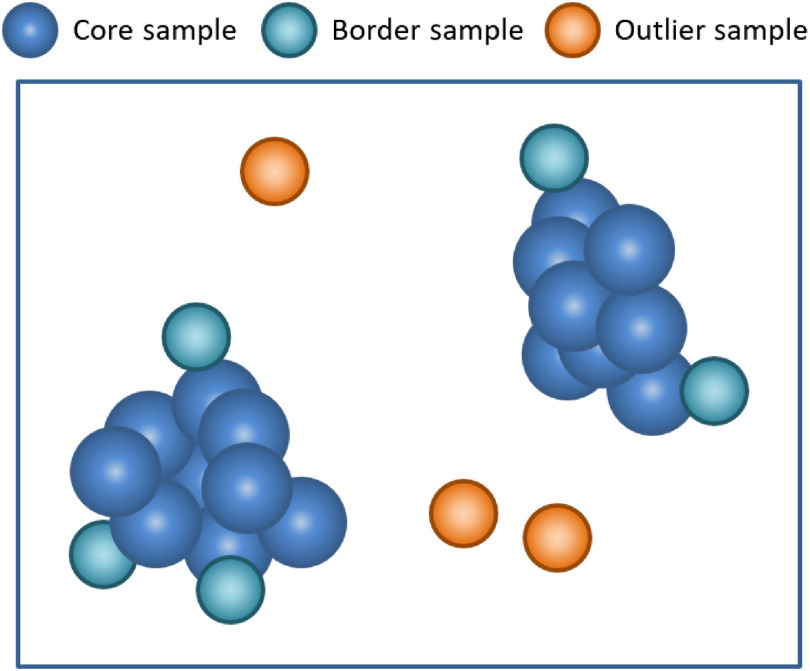
Outlier sample detection principle diagram based on DBSCAN.

DBSCAN^18^ is adopted to mark image samples with a high sample density of the optimal neighborhood radius as cell images. Then, it labels the rest of the outlier image samples as noise samples. These outlier samples, which are sparse and significantly different from the cell sample groups, are removed from training sample set. To group the dataset, DBSCAN requires two parameters, namely the epsilon radius R and minimum number of neighbor points (MinPts). The principle of DBSCAN outlier sample detection is shown in Fig. 3. First, image samples that contain at least MinPts neighbors within the area enclosed by R are labeled as core samples. Then, the samples that lie within an R radius of a core sample, but not being core samples themselves, are labeled as border samples. Finally, the rest of the objects that fall neither in the category of core samples or of border samples are the outlier samples to be rejected.

DENCLUE^19^ nominates a sample as a cell image by defining the degree of closeness of the image to a dense group. The density factors of each sample are calculated by a kernel density function and then summed to be the total density model of the complete dataset. The local maximum of the total density function is the center sample of each cluster. And the samples whose density values are too small and cannot be connected to the cluster center samples are defined as noise samples and are discarded.

LOF^20^ assigns each sample a unique outlier value. The strength of the algorithm is its capability to find the local outliers. It assigns an outlier score to each of the objects depending on the local density of the neighborhood of the concerned object. A sample that is surrounded by a neighborhood with low density is categorized as an outlier, whereas an object with a large number of neighbors is categorized as a cell sample.

To compare the performance of outlier detection methods based on clustering, we randomly select 50% of cell images from both groups as the training set and the remaining 50% as the test set. The training images are checked by three outlier detection methods, respectively. The classification accuracies of XGBoost classification models fitted accordingly are recorded in Table 1 and compared with the performance of the original training set. It is evident from the given table that the most effective method is DBSCAN, which increases classification accuracy by over 1.4%. In terms of runtime, the DBSCAN algorithm also takes the lead. Therefore, we apply DBSCAN to remove the outlier cell images from the training set to establish cell image libraries.

**Table 1:** Comparison of outlier detection methods.

	DBSCAN	DENCLUE	LOF	None
Accuracy	0.9716	0.9522	0.9691	0.9574
Runtime (s)	88.72	494.30	544.49	—

## 4 Feature Extraction and Selection

Most machine learning algorithms except neural networks need extracted features as input to train models and test samples. The suitable features could help classification models operate accurately with low time cost. Four feature extraction methods producing high-dimensional features (feature dimension usually over 1000) are tested to find the most efficient feature for obtained cell images: Gabor wavelet, principal components analysis (PCA), local binary pattern (LBP), and histograms of oriented gradients (HOG). They represent profile feature, full-image dimension reduction feature, local texture feature, and global texture feature, respectively. Furthermore, the performance of fused features also interests us. However, calculating fused high-dimensional features would be time-consuming for the classification procedure because much deeper learning depth would be demanded. Therefore, we present two low-dimensional features, gray histogram and cell size, to combine with the high-dimensional features above. The cell size feature represents the height and width of the cell area in each sample image.

**Fig. 4:**
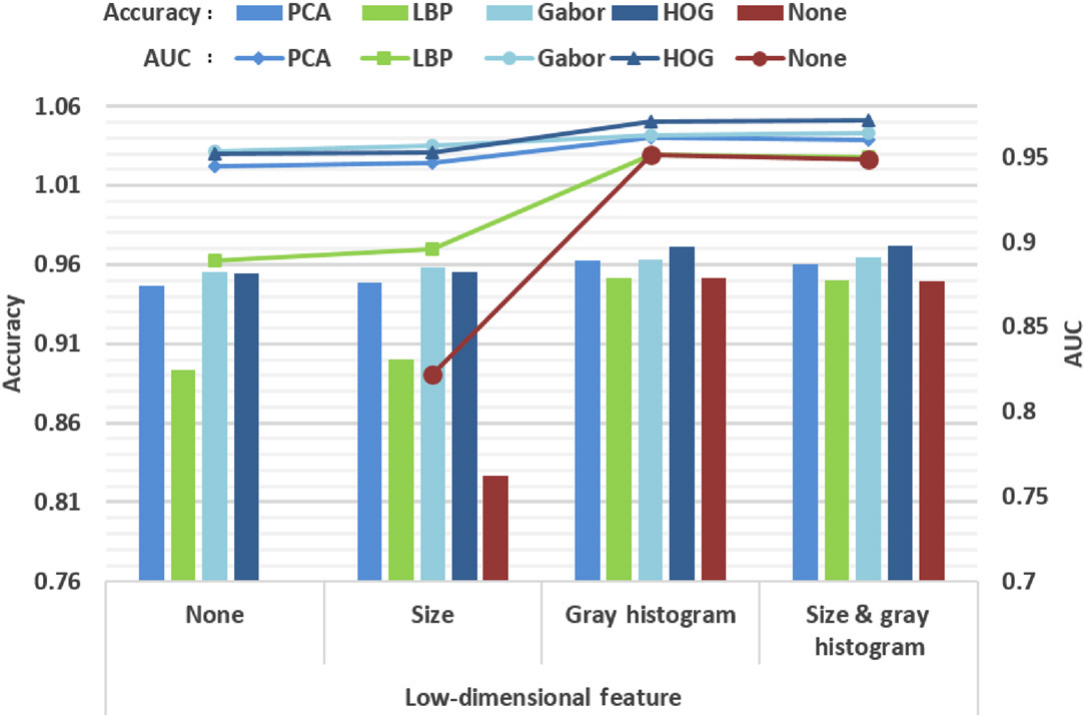
The classification accuracy and AUC of high-dimensional features fused with low-dimensional features.

To compare the performance of four high-dimensional features and their combined features, 50% of cell images are picked randomly from both groups as the training set for the experiment and the remaining 50% as the test set. After features being extracted, different features fit different XGBoost models for classification. Finally, the features of the test set are calculated and grouped by the XGBoost models, respectively. The computing time of feature extraction of the test set is shown in Table 2 and the classification accuracy is given in Fig. 4.

**Table 2:** Runtime comparison of feature extraction methods.

	High-dimensional feature				Low-dimensional feature	
	PCA	LBP	Gabor	HOG	Gray histogram	Size
Feature extraction time (s)	912.16	49.96	964.64	111.03	15.9	45.92
Classification time (s)	0.243	1.388	2.38	3.787	0.06	0.04

It can be seen from the results that, compared to cell size feature, gray histogram better enhances both the accuracy and area under the receiver operating characteristic curve (AUC) of all four high-dimensional features and even beats the fusion of both size and gray histogram occasionally. Moreover, gray histogram also has the lowest time cost. Therefore, gray histogram is implemented for feature combination. Then, among the four high-dimensional methods, Gabor and PCA feature extractions take a much longer time because of convolution operations and large-scale matrix calculation, respectively. LBP takes a minimum amount of time although it is missing the accuracy. With the supplement of gray histogram, the accuracy of HOG is greater than the other features. Therefore, we select the HOG combining gray histogram (HOG-gray) feature for the high-throughput phenotyping framework.

## 5 Classification Based on XGBoost

**Fig. 5:**
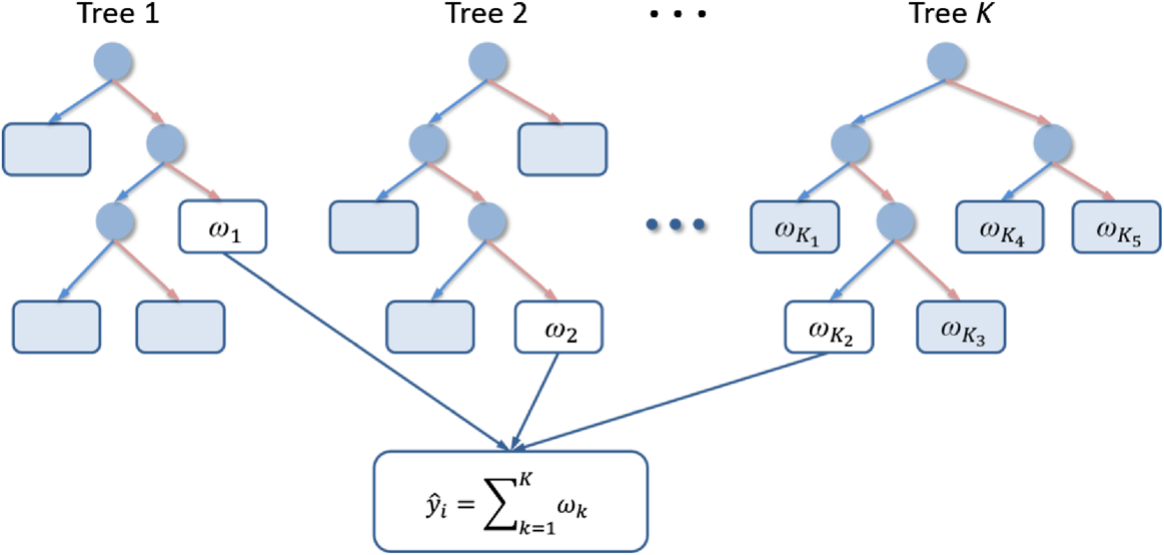
The iteration diagram of XGBoost.

XGBoost^15^ is a promotion method of the traditional gradient boosting decision tree (GBDT). The GBDT iterative decision tree model consists of multiple decision trees. Each iteration brings about a new tree. The final output is formed by the cumulative results of the various decision trees as is shown in Fig. 5.

XGBoost performs a second-order Taylor expansion of the loss function to iterate and calculate the leaf node weights ω of the new tree K. In addition, a regularization term is added to the loss function to control the complexity of the model and prevent it from overfitting. Therefore, XGBoost performs better in terms of modeling effects, training efficiency, massive parallelism, and quadratic convergence. Here, assuming that K trees are produced, the expression of the predicted value yi^ of $x_{i}$ is

yi^=∑k=1Kfk(xi), (1)

where $f_{k}$ is the K’th regression tree. Here, the objective function of iteration is

L(k)=∑i=1nl[yi,yi^k−1+fk(xi)]+Ω(fk), (2)

where l is the loss function and $\Omega (f_{k})$ is the regularization term. Define gi=∂y^(k−1)l(yi,y^(k−1)), hi=∂y^(k−1)2l(yi,y^(k−1)), $I_{j}=\{i|q(x_{i})=j\}$, and $\Omega (f_{k})=\gamma T+\frac{1}{2}\lambda {\left\|\omega \right\|}^{2}$, where q(x) represents the decision tree structure and T represents the number of leaf nodes. As the above terms are substituted into Eq. (2) and second-order Taylor expansion is performed, the objective function is simplified to

$$L(k)=\overset{T}{\underset{j=1}{\sum }}[(\underset{i\in I_{j}}{\sum }g_{i})\omega_{j}+\frac{1}{2}(\underset{i\in I_{j}}{\sum }h_{i}+\lambda )\omega_{j}^{2}]+\gamma T.$$ (3)

The optimal leaf weights are found by deriving Eq. (3) with respect to $\omega_{j}$:

$$\omega_{j}=-\frac{G_{j}}{H_{j}+\lambda },$$ (4)

where $G_{j}=\underset{i\in I_{j}}{\sum }g_{i}$ and $H_{j}=\sum_{i\in I_{j}}h_{i}$.

Three other classification algorithms mentioned above are applied to phenotype same cell images as a comparison: SVM, LR, and convolutional neural network (CNN). SVM and LR are popular machine learning methods in image detection. In addition, with the recent research boom in neural network algorithms, most studies related to image classification of time-stretch imaging systems have focused on neural network algorithms.^13^^,^^21^^,^^22^

The SVM^23^ model has been applied extensively for classification, image recognition, and bioinformatics. It maps image features from low dimension to high dimension by a kernel to create a hyperplane in feature space with the largest interval between sample groups. LR^24^ is a generalized linear model. The mapped value by LR of input features, which is between (0, 1), is considered to be a probability of the sample belonging to the positive sample set.

CNN with convolutional layers can directly convolve with image data to read images and extract their features, which is suitable for images with complex backgrounds. In this paper, we apply AlexNet,^25^ which is a recently developed deep neural network. We optimized the parameters of AlexNet to reduce its complexity for better calculation speed while maintaining its classification accuracy.

**Fig. 6:**
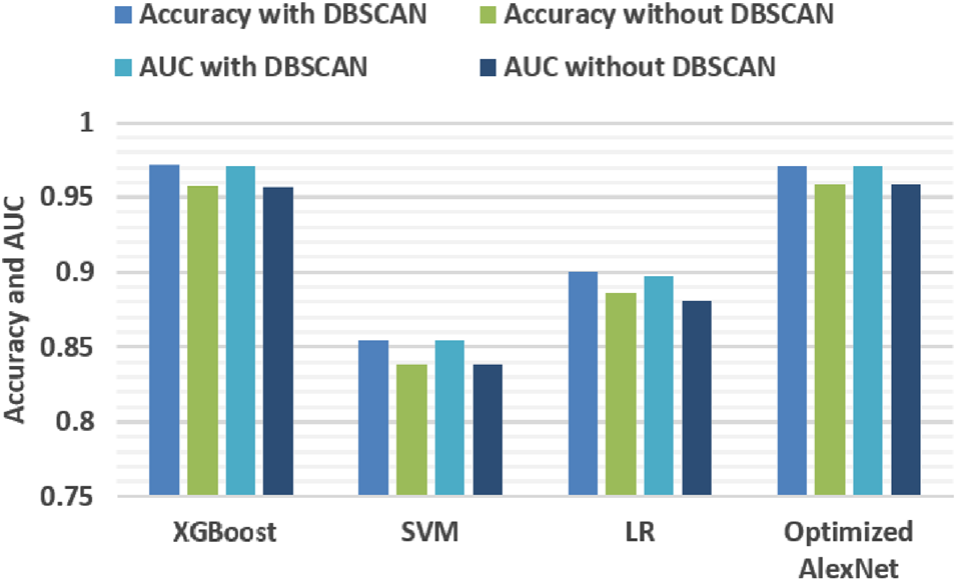
The classification accuracy and AUC of classification algorithms with/without DBSCAN preprocessing.

**Fig. 7:**
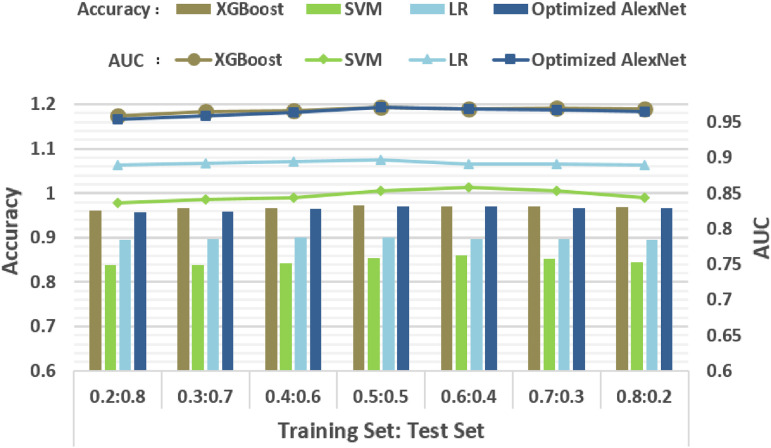
The classification accuracy and AUC of classification algorithms under different size ratios between the training set and the test set.

To compare the performance of XGBoost against the other classification algorithms, the HOG-gray features of the training set are drawn out to fit the SVM, LR, and XGBoost models. The optimized AlexNet model is trained by a raw training image dataset. Then, we check these four models with the test dataset or features accordingly. We repeat the classification steps seven times on different training set sizes to verify their robustness. Moreover, the composition of training sets is also explored by adjusting the ratio of group 1 samples from 20% to 80%. The computing time of testing and the classification accuracy of each classification algorithm are shown in Figs. 6 and 7 and Table 3. Figure 6 also shows the classification accuracies when DBSCAN outlier detection is employed to remove the outlier samples in advance.

**Table 3:** Runtime of classification methods.

	XGBoost	SVM	LR	Optimized AlexNet
HOG-gray extraction (s)	111.03	111.03	111.03	—
Image classification (s)	3.589	86.84	2.72	165.6
Total runtime (s)	114.62	197.87	113.75	165.6

As can be seen from Table 3, compared with the high efficiency of XGBoost, SVM and optimized AlexNet take longer time to label the samples. Since AlexNet is a deep network, the multilayer convolution operation results in high computational complexity and a large deal of memory accesses. If the reading of a single input value or writing of a single output value is recorded as “one memory access,” the total number of memory accesses of optimized AlexNet to classify one cell image (size 191×191×1  pixels) is $3.58\times 10^{8}$. By contrast, the total memory access number of classifying one cell image with HOG-gray-fitted XGBoost classification is $3.63\times 10^{7}$. The large-scale matrix operation cost of SVM is proportional to the sample set size, namely, the larger sample set is, the higher the computational cost is. In terms of classification accuracies, XGBoost and optimized AlexNet take the top, while LR and SVM lag behind. This experiment proves the previous inference that the performance of general machine learning methods is not inferior to deep learning methods in this specific application. Compared with neural network, which endures high computational complexity, XGBoost consisting of weak classifiers achieves high speed in computation that advances when operating under specific and simple scenarios. Therefore, XGBoost classification that has reached the AUC of 0.972 and recognizes 2958  cells/s phenotypes the samples most accurately with celerity and is adopted as the key structure of our framework.

In addition, the DBSCAN outlier detection algorithm has improved the accuracies of all four models by 1% to 2% by removing noise samples from training sets, which also enhances the robustness of the constructed framework. In addition, we experiment on different compositions of training sets to further the performance of the classification algorithms. It is evident from Fig. 7 that different training sample set sizes have little influence on the accuracies of these algorithms, which proves that our algorithm remains robust on small training sample sets. It can also be concluded that algorithms’ accuracies mostly no longer increase when the training set size of each sample group reaches around 5000 samples. That is to say, for future optofluidic imaging experiment, the reasonable size to construct cell image training libraries is about 5000 samples of each type.

**Fig. 8:**
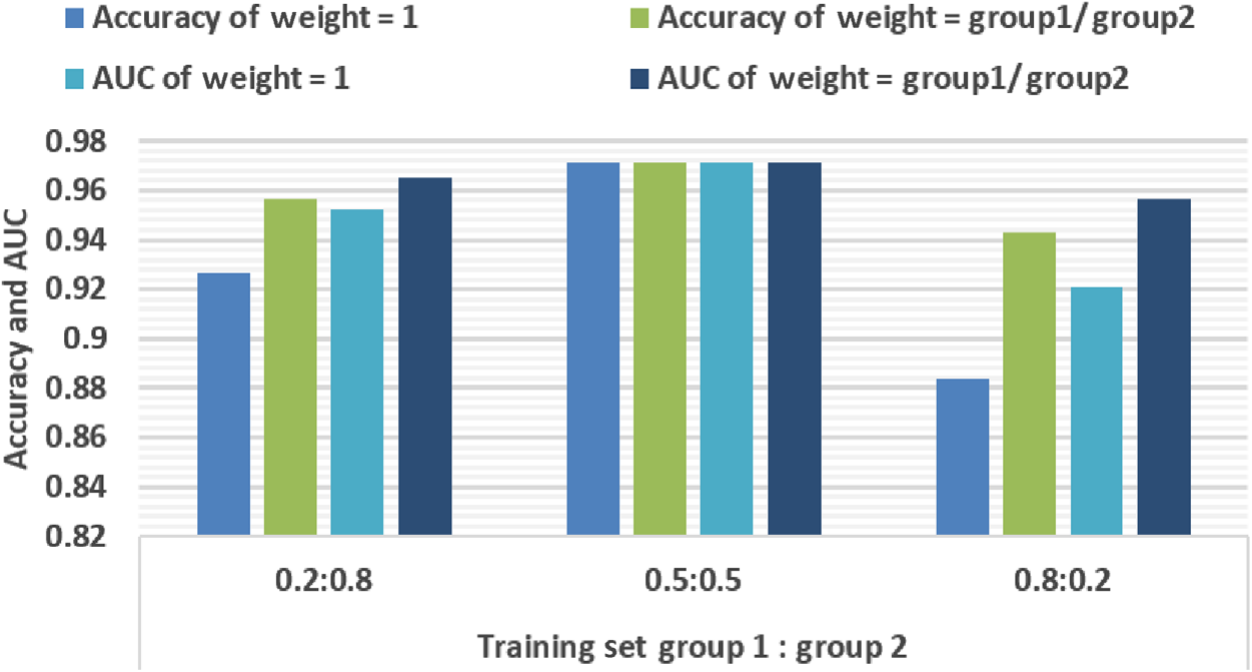
The classification accuracy and AUC of XGBoost under different ratios between the training set size of group 1 and group 2 with/without weight adjusting.

To further guide the following optofluidic time-stretch studies, an experiment on the effect of sample balance on classification accuracy is conducted. We fit HOG-gray features to XGBoost models under three different size ratios between training group 1 and training group 2. Then, the sample weights are set accordingly. The classification results are shown in Fig. 8. As one can see, adjusting sample weights is an effective solution to unbalanced training sets, which promotes their classification accuracies significantly. However, balanced training samples’ performance is still better than unbalanced ones even if weight-adjusting is applied.

## 6 Generalization Ability of the Proposed Framework

**Fig. 9:**
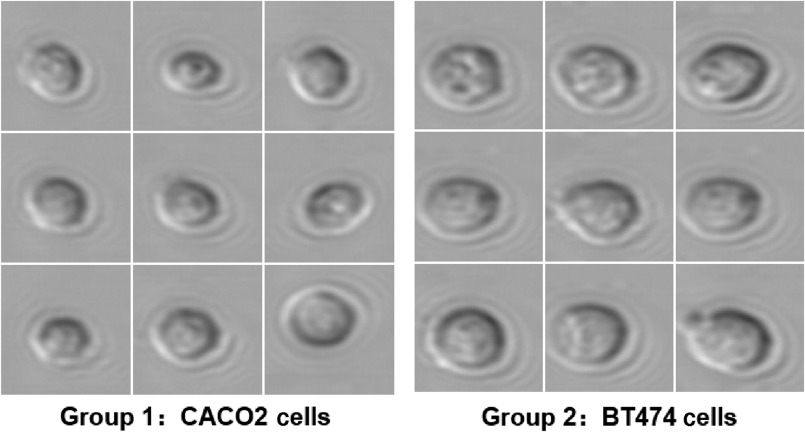
Image libraries of CACO2 and BT474 cells under optofluidic time-stretch microscope.

A fast efficient three-step framework for high-throughput optofluidic time-stretch microscopy consisting of DBSCAN outlier detection, HOG-gray feature extraction, and XGBoost classification is developed according to the experiment results above. To verify the generalization ability of the proposed algorithm, we acquire another set of cell images obtained by an optofluidic time-stretch imaging cytometer. As shown in Fig. 9, group 1 consists of CACO2 cells and group 2 consists of BT474 cells. A total of 2324 cell images (1202 CACO2 cell images and 1122 BT474 cell images) are collected at a throughput of 500  cells/s. We randomly select 50% of cell images from both groups as the training set and the remaining 50% as the test set. HOG-gray, PCA-gray, Gabor-gray, and LBP-gray features are extracted and compared. Then SVM, LR, and XGBoost models are fit by HOG-gray features, and optimized AlexNet is fit by test images. The accuracy and processing time of these algorithms are recorded in Tables 4 and 5. As shown in Table 4, HOG-gray remains a high-level performance. However, PCA-gray’s accuracy is equivalently high and even faster. The reason for this is that the matrix operation scale of PCA decreases on small test datasets and reduces runtime significantly. Therefore, PCA-gray is an alternative choice of feature extraction for small-scale samples. In Table 5, the performance of XGBoost is shown to be outstanding as expected.

**Table 4:** Comparison of feature extraction methods.

	PCA-gray	LBP-gray	Gabor-gray	HOG-gray
Accuracy	0.9205	0.9048	0.9199	0.9267
Runtime (s)	5.39	7.51	109.71	14.52

**Table 5:** Comparison of classification methods.

	Accuracy with DBSCAN	Accuracy without DBSCAN	Runtime of model classification (s)	Total runtime (s)
XGBoost	0.9267	0.9114	0.406	14.93
SVM	0.8471	0.8289	14.08	28.60
LR	0.8739	0.8524	0.615	15.14
Optimized AlexNet	0.9239	0.9090	31.78	31.78

## 7 Conclusions

We proposed an intelligent cell phenotyping framework for high-throughput optofluidic time-stretch microscopy based on an XGBoost algorithm and tested its performance by classifying acquired drug-treated and untreated cell images. Results show that DBSCAN outlier detection, HOG-gray feature extraction, and XGBoost classification have the highest level of accuracy and speed in comparison with other algorithms under specific constraints. The generalization ability of this proposed framework is verified on another set of cell images, and robustness is proved on small-scale training sets. PCA-gray feature is an optional choice for small-scale samples. Therefore, we propose this three-step recognition framework based on XGBoost as a promising solution to processing the large amount of image data of optofluidic time-stretch microscopy accurately and rapidly. The experiment results also offer guidance for training sample set size to construct suitable time-stretch cell image libraries. This work provides a foundation for future cell sorting and clinical practice of high-throughput imaging cytometers.
